# Solid Lipid Curcumin Particles Induce More DNA Fragmentation and Cell Death in Cultured Human Glioblastoma Cells than Does Natural Curcumin

**DOI:** 10.1155/2017/9656719

**Published:** 2017-11-19

**Authors:** Panchanan Maiti, Abeer Al-Gharaibeh, Nivya Kolli, Gary L. Dunbar

**Affiliations:** ^1^Field Neurosciences Institute Laboratory for Restorative Neurology, Central Michigan University, Mt. Pleasant, MI 48859, USA; ^2^Program in Neuroscience, Central Michigan University, Mt. Pleasant, MI 48859, USA; ^3^Department of Psychology, Central Michigan University, Mt. Pleasant, MI 48859, USA; ^4^Field Neurosciences Institute, St. Mary's of Michigan, Saginaw, MI 48604, USA; ^5^Department of Biology, Saginaw Valley State University, Saginaw, MI 48710, USA

## Abstract

Despite recent advancements in cancer therapies, glioblastoma multiforme (GBM) remains largely incurable. Curcumin (Cur), a natural polyphenol, has potent anticancer effects against several malignancies, including metastatic brain tumors. However, its limited bioavailability reduces its efficiency for treating GBM. Recently, we have shown that solid lipid Cur particles (SLCPs) have greater bioavailability and brain tissue penetration. The present study compares the efficiency of cell death by Cur and/or SLCPs in cultured GBM cells derived from human (U-87MG) and mouse (GL261) tissues. Several cell viability and cell death assays and marker proteins (MTT assay, annexin-V staining, TUNEL staining, comet assay, DNA gel electrophoresis, and Western blot) were investigated following the treatment of Cur and/or SLCP (25 *μ*M) for 24–72 h. Relative to Cur, the use of SLCP increased cell death and DNA fragmentation, produced longer DNA tails, and induced more fragmented nuclear lobes. In addition, cultured GBM cells had increased levels of caspase-3, Bax, and p53, with decreases in Bcl_2_, c-Myc, and both total Akt, as well as phosphorylated Akt, when SLCP, rather Cur, was used. Our *in vitro* work suggests that the use of SLCP may be a promising strategy for reversing or preventing GBM growth, as compared to using Cur.

## 1. Introduction

Glioblastoma multiforme (GBM) is one of the most prevalent, deadliest, and aggressive brain cancers (grade-IV astrocytoma, WHO) affecting millions of people worldwide [1]. It accounts for ~60–70% of gliomas [2] and 15% of primary brain tumors [3], with the median survival time being about 15 months following its initial diagnosis [1]. Despite current advances in existing therapeutic modalities, including surgery, radiotherapy, and chemotherapies, GBM remains incurable. Although the use of chemotherapeutic agents, such as the DNA-alkylating agent, temozolomide (TMZ), provides modest survival benefits for the GBM patient [4–6], these drugs are unable to stop the progression of this disease [7, 8], because GBMs are inherently resistance to TMZ. In search of alternative therapies, several investigators [9–13] have studied the anticancer effects of curcumin (Cur), a natural polyphenol, in human malignancies, including those found in various tissues, such as breast, prostate, colon, liver, and brain.

Curcumin is a bright, yellow-colored pigment, derived from the root of the herb, *Curcuma longa*, a traditional spice from Indian and South Asian countries [14]. Because of its potential inhibitory effects on tumor growth, especially the suppression of cellular transformation and inhibition of cell proliferation, invasion, angiogenesis, and metastatic effects, Cur has been targeted for therapeutic application in several cancers, including GBM [13, 15, 16]. Higher concentration of Cur kills cancer cells and can be used to treat different cancers [17, 18], by generating ROS and disrupting AKT/mTOR signaling [9], inducing apoptotic death [11], inhibiting NF-*κ*B in human neuroblastoma [10]. Similarly, Cur suppresses growth and chemoresistance of cultured U-87MG cells via AP-1 and NF-*κ*B transcription factors [13], induces apoptosis in SH-SY5Y cells through nuclear translocation and activation of p53 [12], and attenuates glioma growth in a syngeneic mouse model by inhibition of the JAK1,2/STAT3 signaling pathway [19].

Unfortunately, because of its poor solubility and instability in physiological fluids, the bioavailability of natural Cur is limited, which is considered one of the major obstacles for delivering the therapeutically significant amounts of Cur for targeting GBM [20, 21]. Different lipidated formulas have been developed by several investigators to increase its solubility and bioavailability for cancer therapy [18, 22, 23]. Recently, solid lipid particles (SLPs), conjugated with Cur (SLCPs; see supplementary Figure S1 available online at https://doi.org/10.1155/2017/9656719), has been characterized by our and other laboratories to increase Cur solubility, stability, and bioavailability *in vitro*, in animal models [24–29, 45–49], as well as clinical studies on Alzheimer's disease [30, 31]. Given this, the present study was designed to compare the mechanistic details of cell death *in vitro* using the cells derived from human (U-87MG) and mouse (GL261) GBM tissues after treatment with Cur and/or SLCP. Our results suggest that SLCP kills more GBM cells than Cur by inducing ROS and other cell death markers, thereby inhibiting cell survival pathways *in vitro.*

## 2. Materials and Methods

### 2.1. Chemicals

Curcumin [purity > 65% (HPLC); catalog number C1386-50G], MTT [3-(4,5-dimethylthiazol-2-yl)-2,5-diphenyltetrazolium], annexin-V staining kit (catalog number ABIN411977), propidium iodide (PI), ethidium bromide (EtBr), agarose, proteinase-K, and other accessory chemicals were procured from Sigma (St. Louis, MO). An in situ BrdU-Red DNA fragmentation assay kit (TUNEL staining kit) was purchased from Abcam (Cambridge, MA, catalog number ab66110). Low melting agarose was from Invitrogen (Grand Island, NY; catalog number 16520050). Cell-ROX® reagent was from Molecular Probe (Grand Island, NY, catalog number C10422). Hoechst 33342 trihydrochloride trihydrate solution was purchased from ThermoFisher Scientific (Grand Island, NY). Solid lipid particles containing Cur (SLCP or Longvida, which contains 26% pure Cur) was gifted from Verdure Sciences (Noblesville, IN). This SLCP consists of high-purity, long-chain phospholipid bilayer and a long-chain fatty acid solid lipid core, which coats the Cur (see supplementary Figure S1). The SLCP has been well characterized by us and others in collaboration with Verdure Sciences [24, 28, 32–34], including clinical studies in Alzheimer's disease [30]. The human origin GBM cell line (U-87MG; catalog number HTB-14), neuroblastoma cell line (SH-SY5Y; catalog number ATCC® CRL-2266™), and N2a cells (catalog number ATCC CCL-131™) were purchased from ATCC (Manassas, VA), whereas mouse GBM cell line (GL261) was procured from DCTD/DTP Tumor Repository at the National Cancer Institute.

### 2.2. Cell Culture

U-87MG and GL261 cell lines were used for this study. Briefly, the U-87MG cells were grown in Eagle's Minimum Essential Medium (EMEM, GIBCO) containing 10% heat-inactivated fetal bovine serum (FBS) and penicillin/streptomycin (pen: 100 I.U./mL; strep: 100 *μ*g/mL). Similarly, the GL261 cells were cultured in Roswell Park Memorial Institute medium-1640 (RPMI-1640), along with 10% FBS and pen (100 I.U./mL) and strep (100 *μ*g/mL). The culture was maintained at 37°C in a humidified atmosphere at 5% CO_2_. Prior to the experiment, the cells were grown either in 60 mm Petri dishes and 96-well plates or on glass coverslips, with fresh EMEM and antibiotics, but without growth factors, depending on the experimental setup. For Cur and/or SLCP permeability study, the N2a cells and mouse primary hippocampal neurons were used. The N2a cells were grown in EMEM, and mouse embryonic-16 (E16) hippocampal neurons were grown in neurobasal media containing B27 supplementation for 7 days, as described previously [35].

### 2.3. Curcumin and/or SLCP Treatment

The solubility and permeability of both Cur and SLCP were investigated in cell cultures and in vivo, as described previously [36]. Because Cur solubilizes best in methanol (28), therefore, the Cur and SLCP were dissolved in pure methanol (100%) and then diluted in Hank's balanced salt solution (HBSS) to obtain its desired concentration before being added to the Petri dish containing the cells. The final methanol concentration was <0.1% (*v/v*).

### 2.4. Cell Viability by MTT Assay

To investigate which concentration and duration of Cur or SLCP treatment kills more GBM, we have conducted a cell viability test, using a MTT [3-(4,5-dimethylthiazol-2-yl)-2,5-diphenyltetrazolium bromide] assay, as described previously [28, 35, 37]. The cells were treated with freshly prepared concentrations of Cur or SLCP (in *μ*M: 1–100) at different time points (in hours: 24, 48, and 72 h). After standardization of toxicity levels, 25 *μ*M of Cur or SLCP was used for all experiments with 24–72 h exposure. The optical density was measured at 570 nm using a Synergy plate reader (Bio-TEK instruments, Winooski, VT). The results of the three independent experiments (6 wells per condition) were normalized to the medium control group and expressed as mean ± SEM.

### 2.5. DNA Fragmentation Study by TUNEL Staining

The TUNEL staining was performed as per manufacturer's instructions [28, 35]. Briefly, U-87MG cells were grown on coverslips in EMEM, without any growth factors for 24 h and then they were treated with Cur or SLCP (25 *μ*M) for 24–72 h. Following treatment, the cells were fixed with 4% paraformaldehyde for 15 min, and then TUNEL staining was performed [28, 35]. Finally, the cells were counterstained with Hoechst 3342 for 5 min at room temperature in the dark and washed thoroughly with distilled water, after which they were mounted on a glass slide with antifading medium. The cells were observed under a fluorescent microscope (Leica, Germany), using appropriate filters (ex/em: 488/576). The red fluorescent signal indicated TUNEL-positive cells. The number of total cells and that of TUNEL-positive cells were counted and expressed as a percentage of the total cell count. Almost two thousand total cells were counted in each group of randomly selected microscopic fields from three independent experiments to obtain a mean value.

### 2.6. Annexin-V/PI Staining for Apoptotic and Necrotic Cell Death

The annexin-V staining was performed, as described previously [28, 38]. Briefly, the U-87MG cells were treated with Cur or SLCP (25 *μ*M), dissolved in methanol, and diluted with HBSS for 24–48 h and then annexin-V-FITC stain was performed, along with counterstaining with PI (500 nM) [28]. The total number of cells and the number of annexin-V-positive cells were counted per microscopic field and expressed as a percentage of dead cells. Approximately, 30 microscopic fields (~1000 total cells) from three independent experimental setups were used for counting.

### 2.7. Single-Cell Gel Electrophoresis (SCGE) or Comet Assay

The comet assay was performed to measure the degree of DNA strand breaks, as described previously [39–41]. Briefly, the U-87MG cells (1 × 10^5^/mL) were grown on Petri-plate in EMEM and treated with Cur or SLCP (25 *μ*M) for 24, 48, and 72 h. After the stipulated period of the treatments, the cells were washed with Dulbecco's PBS (DPBS), scraped, and centrifuged to get a pellet. Then 75 *μ*L of 0.5% low melting agarose (dissolved in PBS and preincubated at 37°C for 30 min before its use) was added to the cell pellet to make a semisolid cell suspension, which was gently added to the top of the agarose layer on the glass slide. Then the cells were lysed and SCGE was performed, followed by counterstaining with EtBr (1 *μ*g/mL) and imaged using a fluorescent microscope (Leica, Germany). The number of total cells and that of comet-positive cells were counted in each microscopic image and expressed as % of comet-positive cells per total cells. At least 1500 total cells were analyzed from three independent experiments to obtain the mean values represented. The % DNA in tail, tail length, tail moment, and olive tail moment was measured using ImageJ software (https://imagej.nih.gov/ij/) using the following formula, as described previously [40, 42, 43]: (i) percentage DNA in head = head fluorescent intensity/(head fluorescent intensity + tail fluorescent intensity) × 100; (ii) percentage DNA in tail = 100 − percentage DNA in head; (iii) tail moment length (*μ*m) = length between the center of the head and the center of the tail; (iv) extent tail moment = tail length × percentage DNA in tail; and (v) olive tail moment = tail moment length × percentage DNA in tail. At least 100 cells in each group from three dependent experiments were used for comet analysis and expressed as mean ± SEM.

### 2.8. Detection of Reactive Oxygen Species (ROS)

Intracellular accumulation of ROS was detected by 2′-7′-dichlorodihydrofluorescein diacetate (DCFH-DA), as described previously [9, 28, 44]. Briefly, the U-87MG cells were grown (1 × 10^5^/well) in EMEM, treated with Cur and/or SLCP (25 *μ*M), and CellRox assay was performed, followed by counterstaining with PI (500 nM). The cells were observed under the fluorescent microscope (Leica, Germany), using appropriate filters (ex/em: 485/520). The presence of green fluorescent signal indicated ROS level. Total fluorescent intensity (arbitrary unit (AU)) of an individual cell was measured using ImageJ software (https://imagej.nih.gov/ij/), and at least 400–500 hundred cells were randomly selected from three independent experiments to obtain a mean value.

### 2.9. Immunocytochemistry

Immunocytochemistry of anti-caspase-3, p53, and c-Myc was performed as described previously [28]. Briefly, the U-87MG cells were grown (1 × 10^5^/well) on a Petri-plate containing glass coverslips in EMEM with pen (100 I.U./mL) and strep (100 *μ*g/mL) for 24 h and then treated with Cur and/or SLCP (25 *μ*M) for another 24 h. Then the cells were incubated with rabbit anti-caspase-3, p53, and c-Myc monoclonal antibodies (1 : 100, see Table 1) for 3 h at 37°C, followed by incubation with respective secondary antibodies (1 : 200) tagged with Texas-red (Molecular Probes, OR) for 1 h at room temperature. Nuclei were stained with Hoechst 33342 (20 mM, ThermoFisher Scientific, Grand Island, NY) and visualized using a fluorescence microscope (Leica, Germany) [28].

### 2.10. DNA Gel Electrophoresis

DNA gel electrophoresis was performed to measure the DNA fragmentation, as described previously [45]. Briefly, U-87MG cells were grown (1 × 10^5^/mL) in EMEM with pen (100 I.U./mL) and strep (100 *μ*g/mL) and kept overnight in T-25 flask and then treated with Cur or SLCP (25 *μ*M) for 24 h. The following day, media and the cells in the flask were scraped and centrifuged at 1200 rpm for 7 min, and from the pellet, the genomic DNA was extracted by the phenol-chloroform extract method and was electrophoresed using 3% agarose gel and staining with EtBr [45]. The gel image was taken using gel documentation system (BioRad, Hercules, CA) using an appropriate filter.

### 2.11. Western Blot

To check the protein levels, Western blot was performed as described previously [28]. Briefly, after the stipulated period of each experiment, the GL261 cells were lysed with cold radio immunoprecipitation assay (RIPA) buffer, along with protease and phosphatase inhibitors. An equal amount of protein, per lane, was loaded and electrophoresed on 10% Tris-glycine gel and transferred to PVDF membrane (Millipore, Bedford, MA). After probing with respective primary (see Table 1) and secondary antibodies, the blots were developed with Immobilon™ Western Chemiluminescent HRP-substrate (Millipore, Billeria, MA). The relative optical density (OD) was measured using ImageJ software (https://imagej.nih.gov/ij/download.html). To ensure equal protein loading in each lane, the blots were stripped and reprobed for *β*-tubulin.

### 2.12. Statistical Analysis

The data were expressed as mean ± SEM. Data were analyzed using one-way analysis of variance (ANOVA), followed by post hoc Tukey HSD (honestly significant difference) test. Probability ≤ 0.05 was considered as statistically significant.

## 3. Results

### 3.1. SLCPs Reduced More Cell Viability than Cur in U-87MG Cells

**Figure 1:**
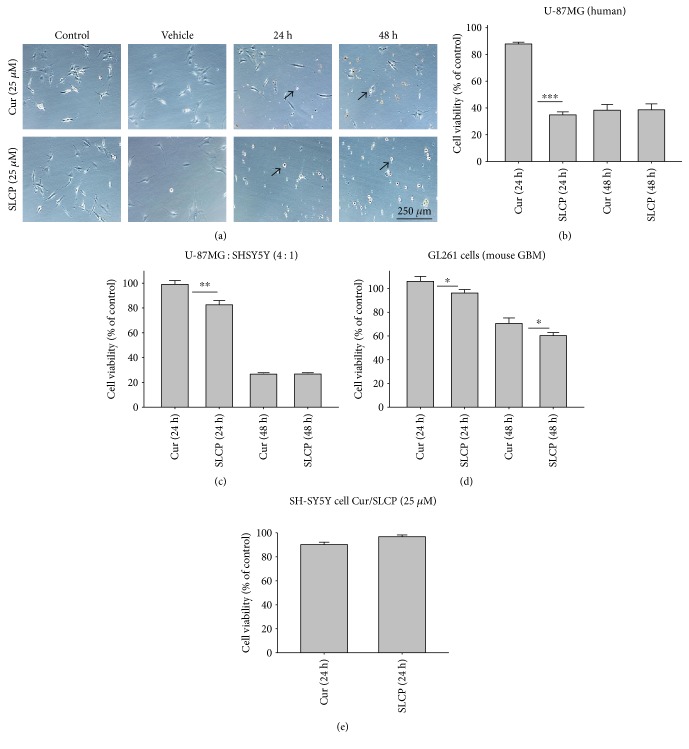
Comparison of morphology and cell viability in U-87MG and GL261 cells after treatment with Cur or SLCP. U-87MG cells were grown in EMEM and pen (100 I.U./mL) and strep (100 *μ*g/mL) for 24 h and then treated with either Cur or SLCP (25 *μ*M) for 24 and 48 h. On the following day, an MTT assay was performed and the % of cell viability was expressed as mean ± SEM from five independent experiments. (a) After 24 and 48 h of treatment, more pyknotic cells were observed with SLCP than with Cur treatment. (b) Similarly, SLCP-treated cells showed less cell viability in comparison to Cur-treated cells after 24 h, but no significant difference was observed between Cur- and SLCP-treated cells after 48 h. (c) A similar phenomenon was observed in the case of U-87MG : SH-SY5Y mixed culture (4 : 1). (d) Cell viability was also significantly decreased in the case of GL261 cells after 24 and 48 h of SLCP treatment relative to Cur treatment. (e) No significant decrease of cell viability was observed in both Cur- and SLCP-treated SH-SY5Y cells. Arrows indicate pyknotic cells. Scale bar indicates 250 *μ*m and is applicable to all images. ^∗^*p* < 0.05, ^∗∗^*p* < 0.01, and ^∗∗∗^*p* < 0.001 in comparison to Cur-treated cells.

To compare the cell death by Cur and SLCP, we have performed MTT reduction assay, which depicts the status of cell viability. We found that SLCPs induced ~66% cell death, whereas it was 11% in the case of Cur-treated cells after 24 h (cell viability for SLCP = 34% and for Cur = 89%, *p* < 0.001) (Figures 1(a) and 1(b)). However, we did not find any difference in cell death after 48 h of their incubation (cell viability for Cur = 38% and for SLCP = 39%) (Figures 1(a) and 1(b)). We also observed a significant difference in cell viability (*p* < 0.05) in a mixed culture of cells derived from human tissue (U-87MG : SH-SY5Y = 4 : 1) after 24 h of Cur and/or SLCP treatment (Figure 1(c)). When we compared the cell viability in the GL261 cells, we observed significantly more cell death (*p* < 0.05) in the case of SLCP after 24 and 48 h of their treatment in comparison to Cur alone (cell viability for SLCP = 60% and for Cur = 70%, after 48 h) (Figure 1(d)). Interestingly, there was no significant change in cell viability in neuroblastoma cells (SH-SH5Y) derived from human tissue after 24 h of Cur and SLCP treatment (Figure 1(e)).

### 3.2. SLCP Induced More TUNEL-Positive (DNA Fragmented) Cells than Cur in U-87MG Cells

**Figure 2:**
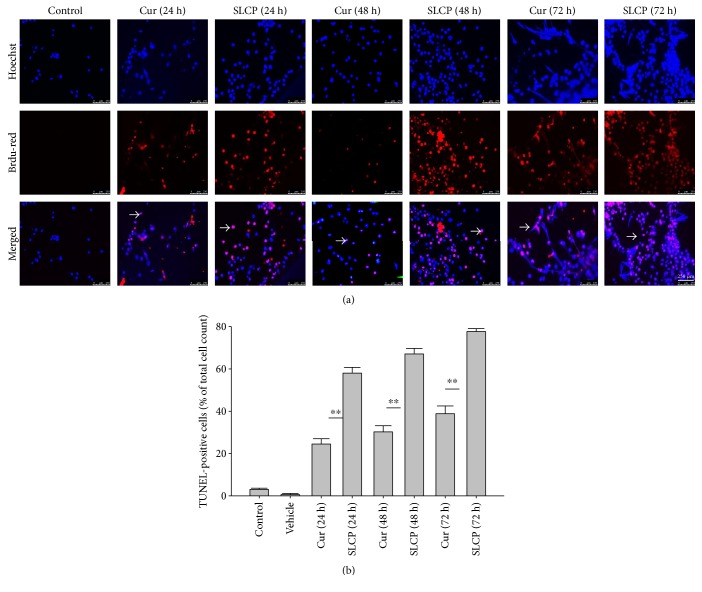
SLCP induced more DNA fragmentation (TUNEL-positive cells) in U-87MG cells than Cur. U-87MG cells were grown in EMEM and pen (100 I.U./mL) and strep (100 *μ*g/mL) for 24 h and then treated with either Cur or SLCP (25 *μ*M) for 24 h, and then TUNEL staining was performed. (a) TUNEL-positive cells (arrow) after treatment with Cur or SLCP for 24–72 h. (b) SLCP-treated cells showed significantly more TUNEL-positive cells in comparison to Cur-treated cells. Scale bar indicates 250 *μ*m and is applicable to all images. ^∗∗^*p* < 0.01 in comparison to Cur-treated, vehicle-treated, or control (untreated) cells.

In situ BrdU-Red DNA fragmentation or TUNEL staining was performed to investigate the number of DNA-fragmented cells after treatment with Cur or SLCP. We found a significantly increased number of TUNEL-positive cells in the case of SLCP-treated cells in comparison to Cur-treated cells after 24 h (Cur = 24.96%; SLCP = 58.20%; *p* < 0.01), 48 h (Cur = 30.59%; SLCP = 67.16%; *p* < 0.01), and 72 h (Cur = 39.19%; SLCP = 77.67%; *p* < 0.01) (Figures 2(a) and 2(b)).

### 3.3. SLCP Induced More Apoptotic and Necrotic Death than Cur in U-87MG Cells

**Figure 3:**
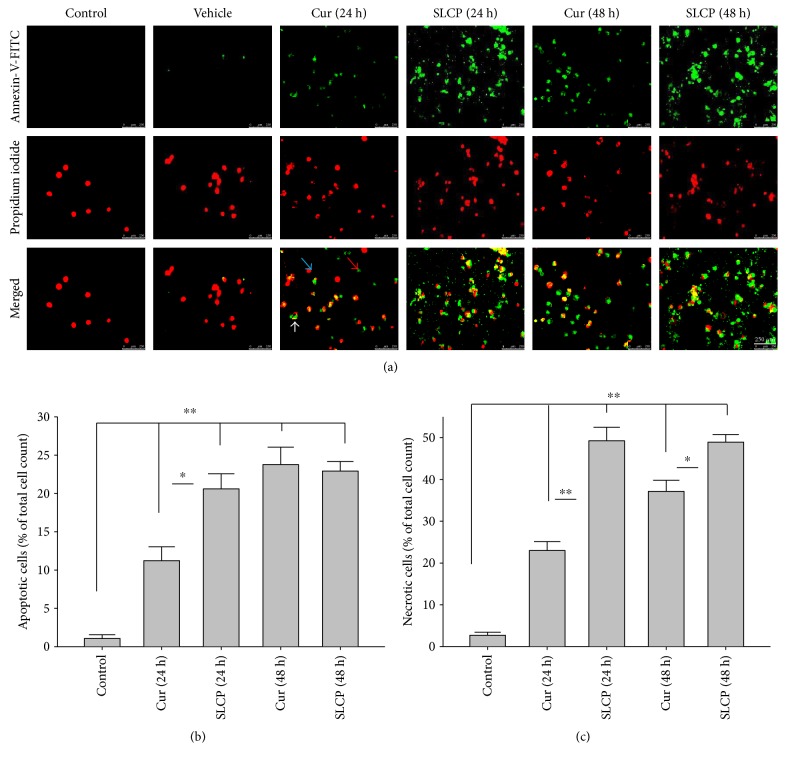
SLCP induced more apoptosis and necrosis in U-87MG cells than Cur. U-87MG cells were grown in EMEM and pen (100 I.U./mL) and strep (100 *μ*g/mL) for 24 h, and then the cells were treated with either Cur or SLCP (25 *μ*M) for 24, 48, and 72 h. The cells were stained with annexin-V, tagged with FITC for detecting apoptotic cell death and counterstaining with PI. The fluorescent microscope (Leica Germany) was used to detect the signal with appropriate excitation/emission filters. (a) Representative images of annexin-V/PI-stained cells after treatment with Cur or SLCP for different time points. (b) The number of apoptotic cells was more in SLCP-treated cells in comparison to that in Cur-treated cells after 24 h of incubation. (c) Similarly, the number of necrotic cells was also more in SLCP-treated cells in comparison to that in Cur-treated cells after 24 and 48 h of incubation. Blue, red, and white arrows indicate normal, apoptotic, and necrotic cells, respectively. Scale bars indicate 250 *μ*m and is applicable to all images. ^∗^*p* < 0.05 and ^∗∗^*p* < 0.01 in comparison to Cur-treated cells and vehicle or control (untreated) cells.

One of the aims of this study was to investigate the type of cell death following treatment of Cur or SLCP. We observed that both Cur and SLCP induced apoptosis and necrosis in U-87MG cells (Figure 3(a)). The number of apoptotic death was significantly higher in the case of SLCP-treated cells (Figure 3(b)) at 24 h than Cur-treated cells (Cur = 13.57%; SLCP = 23.34%; *p* < 0.05). Similarly, SLCP induced significantly more necrotic death than did Cur after 24 h (Cur = 22.99%; SLCP = 49.99%; *p* < 0.01) and 48 h (Cur = 37.14%; SLCP = 49.92%; *p* < 0.05) of incubation **(**Figure 3(c)**)**.

### 3.4. SLCP Causes Increased Nuclear Lobe Formation than Cur in U-87MG Cells

**Figure 4:**
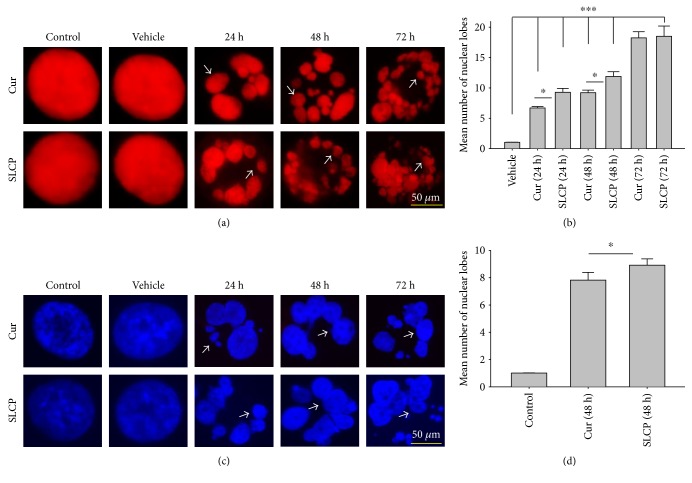
Nuclear morphology in U-87MG cells after treatment with Cur or SLCP. U-87MG cells were grown in EMEM and pen (100 I.U./mL) and strep (100 *μ*g/mL) for 24 h, and then the cells were treated with either Cur or SLCP (25 *μ*M) for 24–48 and 72 h, followed by stained with PI (a) and Hoechst 3342 (c). The images were taken with a fluorescence microscope (Leica, Germany) using 100x objectives (total magnification 1000x). (a, c) Representative images of nuclear morphology after treatment with Cur or SLCP. (b) The mean number of nuclear lobes was significantly more in the case of SLCP-treated cells in comparison to that in Cur-treated cells after 24 and 48 h of treatment. (d) Similar pattern was observed in the case of Hoechst 3342-stained cells after 48 h of Cur or SLCP treatment. Arrows indicate fragmented nuclear lobe. Scale bars indicate 50 *μ*m and is applicable to all images. ^∗^*p* < 0.05 in comparison to Cur-treated cells and ^∗∗∗^*p* < 0.001 in comparison to vehicle or control (untreated) cells.

DNA fragmentation is one of the important phenomena observed in cell death. The fragmentation of DNA causes the formation of several nuclear lobes, depending on the degree of fragmentation and duration of drug treatment. We found a significant increase in the number of nuclear lobes in the SLCP-treated than in Cur-treated cells after 24 and 48 h (*p* < 0.05) (Figures 4(a) and 4(b)). Similar phenomena were also observed when nuclear morphology was studied by Hoechst 33342 (Figures 4(c) and 4(d)).

### 3.5. SLCP Induced More Comet-Positive Cells than Cur in U-87MG Cells

**Figure 5:**
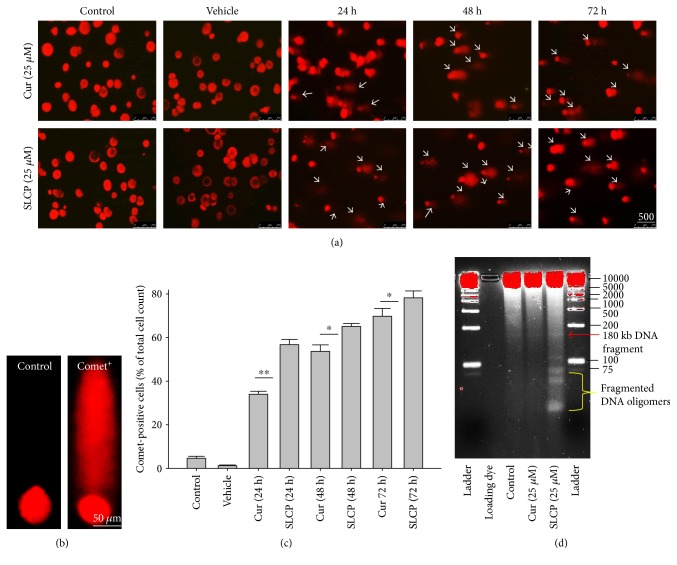
SLCP induced more DNA fragmentation than Cur in U-87MG cells as revealed by comet assay (SCGE) and gel electrophoresis. U-87MG cells were grown in EMEM and pen (100 I.U./mL) and strep (100 *μ*g/mL) for 24 h, and then the cells were treated with either Cur or SLCP (25 *μ*M) for 24–72 h. Cells were lysed in lysis solution and run in electrophoretic chamber for 30 min, and the fragmented DNA tail was stained with EtBr. The images were taken by a fluorescence microscope (Leica, Germany) with appropriate filters. (a) Representative images showed fragmented DNA tail (comet) after treatment with Cur or SLCP for different time points. (b) Typical morphology of normal cell and comet-positive cell after staining with EtBr. (c) The number of comet-positive cells was more in the case of SCLP- than Cur-treated cells after 24–72 h. (d) DNA gel electrophoresis showed more DNA fragmentation in SLCP-treated cells than Cur-treated cells after 24 h. Arrows indicate comet-positive cells. Scale bar indicates 500 *μ*m in “A” and 50 *μ*m in “B” and is applicable to all images in each of these figures. ^∗^*p* < 0.05 and ^∗∗^*p* < 0.01 in comparison to Cur-treated cells.

SCGE or comet assay is one of the gold standard methods to investigate the degree of DNA fragmentation *in vitro*. The number of comet-positive cells was significantly increased in SLCP-treated cells after 24 h (Cur = 34.00; SLCP = 56.76; *p* < 0.01), at 48 h (Cur = 53.64; SLCP = 65.11; *p* < 0.05), and 72 h (Cur = 69.78; SLCP = 78.21; *p* < 0.05) in comparison to Cur-treated cells (Figures 5(a) and 5(c)).

### 3.6. DNA Gel Electrophoresis

To visualize the degree of DNA fragmentation by Cur or SLCP treatment, we have performed DNA gel (3%) electrophoresis. We observed that SLCP produced small DNA fragments, including lower nucleotides oligomers, which was less than 100 kilobase pair (kb), whereas similar fragmentation was not observed in the case of Cur-treated or control cells (Figure 5(d)).

### 3.7. Degree of DNA Fragmentation Was More in SLCP than in Cur-Treated U-87MG Cells

**Figure 6:**
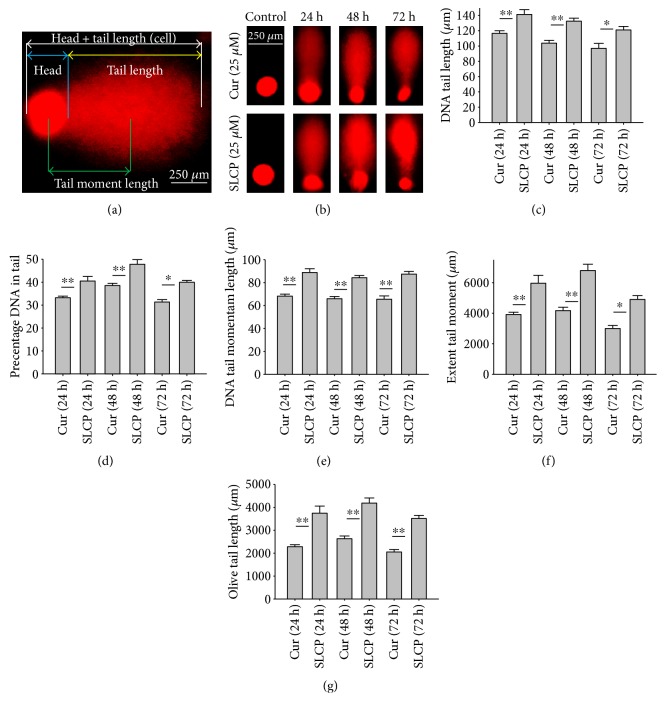
Comet assay (SCGE) in U-87MG cells after treatment with Cur or SLCP. U-87MG cells were grown in EMEM and pen (100 I.U./mL) and strep (100 *μ*g/mL) for 24 h, and then the cells were treated with either Cur or SLCP (25 *μ*M) for 24 h. (a) Different parameters, such as nuclear head fluorescent intensity, fragmented DNA tail intensity, tail length, tail moment length, % DNA in tail, extended tail length (*μ*m), and olive tail length (*μ*m) were measured after treatment with Cur or SLCP. (b) Representative comet-positive cells after treatment with Cur and/or SLCP for 24–72 h. Note that SLCP-treated cells showed greater DNA tail (c), % DNA in tail (d), DNA tail moment length (e), extent tail moment, and olive tail length after 24–72 h in comparison to Cur-treated cells. Scale bars indicate 250 *μ*m and is applicable to all images. ^∗^*p* < 0.05 and ^∗∗^*p* < 0.01 in comparison to Cur-treated cells.

One of the focus of this study was to investigate the degree of DNA fragmentation after treatment with Cur or SLCP. Based on the fluorescent intensity of head and fragmented DNA tail, DNA tail length, DNA tail moment length (Figure 6(a)), the % DNA in tail, extended DNA tail length (*μ*m), and olive tail length (*μ*m) have been calculated (Figures 6(c), 6(d), and 6(e)). SLCP treatment showed significantly higher DNA tail length (Figure 6(c)) and % of DNA in tail (Figure 6(d)) after 24 h (*p* < 0.01), 48 h (*p* < 0.01), and 72 h (*p* < 0.05) in comparison to Cur-treated cells. Similarly, DNA tail moment length (*μ*m) was also significantly higher (*p* < 0.01) after 24, 48, and 72 h of SLCP-treated cells in comparison to Cur-treated cells (Figure 6(e)). In addition, extended tail moment and olive tail length were also significantly higher in the case of SLCP-treated cells after 24 and 48 h (*p* < 0.01) of treatment, in relative to Cur-treated cells (Figures 6(f) and 6(g)).

### 3.8. SLCP Increased ROS Level Greater than Cur in U-87MG Cells

**Figure 7:**
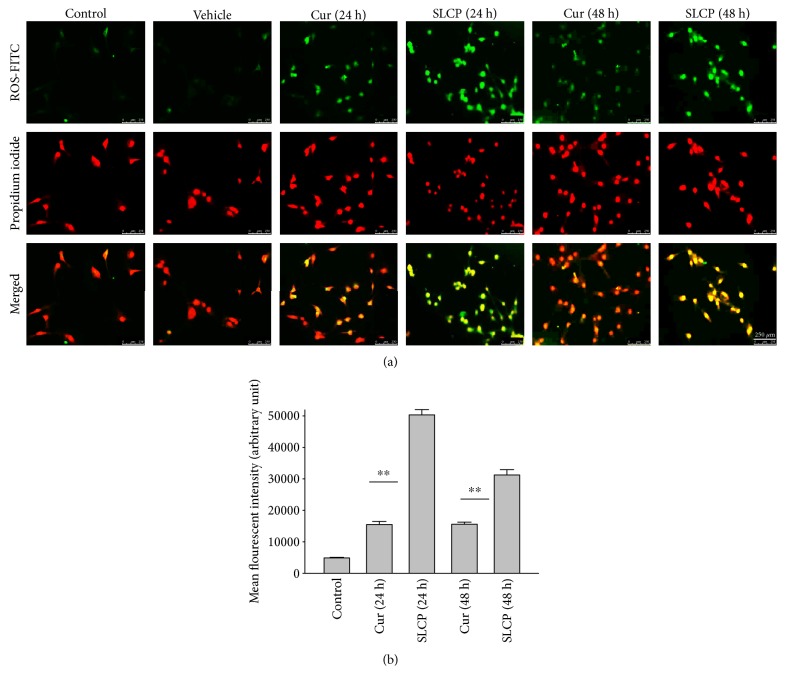
SLCP-treated cells produced more ROS than Cur. U-87MG cells were grown in EMEM and pen (100 I.U./mL) and strep (100 *μ*g/mL) for 24 h and then treated with either Cur or SLCP (25 *μ*M) for 24 and 48 h and labeled with CellROX reagent and counterstaining with PI. (a) The images were taken using a fluorescent microscope (Leica, Germany) with appropriate excitation/emission filters. Green fluorescent signal indicates ROS production. (b) SLCP-treated cells showed more ROS production after 24 and 48 h of its incubation in comparison to Cur. Scale bar indicates 250 *μ*m and is applicable for all the images. ^∗∗^*p* < 0.01 in comparison to Cur-treated cells.

To investigate the mechanism of cell death, the U-87MG cells were treated with Cur or SLCP (25 *μ*M) for 24 or 48 h and stained with CellROX oxidative stress reagents. We observed that ROS levels were significantly increased by SLCP exposure after 24 h [in AU: Cur = 15493.99; SLCP = 50297.27; *p* < 0.01] and 48 h [in AU: Cur = 15600.00; SLCP = 31228.32; *p* < 0.01] in comparison to Cur-treated cells (Figures 7(a) and 7(b)).

### 3.9. SLCP Increased Cell Death Markers and Reduced Cell Survival Markers More than Cur in GL261 and U-87MG

**Figure 8:**
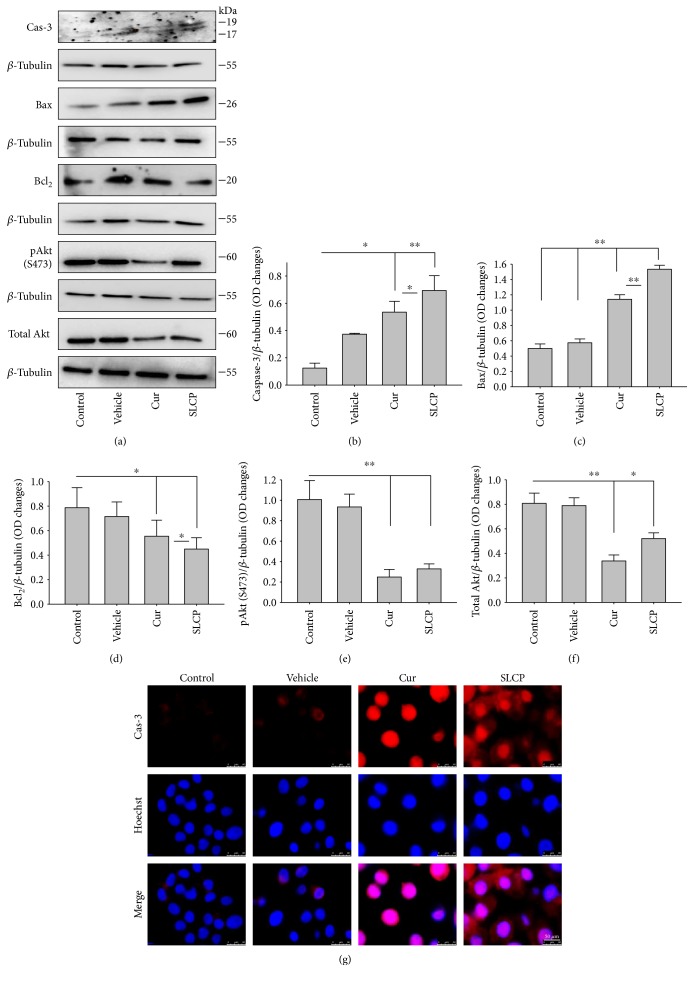
SLCP-treated cells induced greater cell death markers and decreased more cell survival marker than Cur. (a–f) Cell survival markers (Akt, p-Akt, and Bcl_2_) were significantly reduced, and cell death markers (caspase-3 and Bax) were significantly increased in SLCP-treated cells in comparison to Cur-treated cells. (g) Immunocytochemistry with U-87MG cells showed an increase in caspase-3 immunofluorescence in both Cur- and SLCP-treated cells. Scale bar indicates 50 *μ*m and is applicable to other images. ^∗^*p* < 0.05 and ^∗∗^*p* < 0.01 in comparison to control (untreated), vehicle, and Cur-treated groups.

We have investigated cell death and cell survival markers from GL261 cells to compare the cell death mechanism after treatment with Cur and/or SLCP. Our Western blot (Figures 8(a) and 8(b)) and immunofluorescence (Figure 8(g)) results showed an increase in active caspase-3 (*p* < 0.05) in the SLCP-treated group, in comparison to Cur-treated cells. Similarly, Bax level was also significantly higher (*p* < 0.01) in the SLCP-treated group, in comparison to Cur-treated cells (Figures 8(a) and 8(c)). In contrast, Bcl_2_ level was significantly lower (*p* < 0.05) in the case of SLCP-treated cells in comparison to Cur-treated cells (Figure 8(d)). Although total Akt and phosphorylated-Akt were significantly decreased (*p* < 0.01) from untreated cells, we did not find any significant difference between the Cur- and SLCP-treated groups (Figures 8(a), 8(e), and 8(f)).

### 3.10. Increased p53 and Decreased c-Myc Levels Were Observed in Cur- and/or SLCP-Treated GL261 and U-87MG Cells

**Figure 9:**
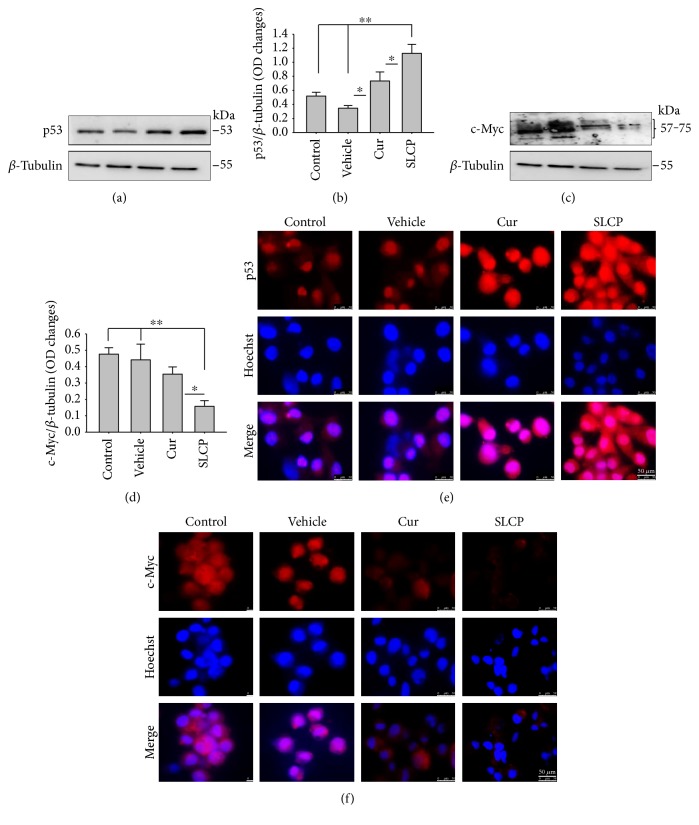
SLCP increased p53 and decreased c-Myc levels greater than Cur in vitro. (a–d) Western blot data showed that SLCP increased p53 and decrease c-Myc levels significantly more in GL261 cells in comparison to Cur-treated or untreated cells. Similarly, immunofluorescence signal of p53 was increased (e) and c-Myc was decreased (f) more in SLCP or Cur-treated U-87MG cells in comparison to untreated cells. Scale bar indicates 50 *μ*m and is applicable to other images. ^∗^*p* < 0.05 in comparison to Cur-treated cells and ^∗∗^*p* < 0.01 in comparison to vehicle and control (untreated) cells.

Western blot analysis from GL261 cells showed significantly increased levels of p53 (Figures 9(a) and 9(b), *p* < 0.05) and decreased levels of c-Myc (Figures 9(c) and 9(d), *p* < 0.01) in both Cur- and SLCP-treated cells, but we found no significant differences between the Cur and SLCP groups in the case of p53 (Figure 9(b)), whereas c-Myc was significantly decreased in SLCP-treated cells in comparison to the Cur-treated group (Figure 9(d)). In addition, our immunofluorescent data from U-87MG cells also showed similar phenomena in both the cases of p53 (Figure 9(e)) and c-Myc (Figure 9(f)).

## 4. Discussion

Standard treatments for GBM have remained ineffective due to the inherent resistance of GBM cells to radiotherapy and chemotherapy, and the invasive propensity of GBM cells limits the effectiveness of surgery [46]. Therefore, finding novel approaches is desperately needed. Recently, several investigators have shown that natural polyphenol Cur attenuates GBM growth, proliferation, and metastasis *in vitro* and in different animal models of glioma [19]. In the present study, we have compared the efficacy of SLCP (a greater permeable solid lipid Cur formula) and natural Cur on GBM cell lines derived from human (U-87MG) and mouse (GL261) tissues. We found that SLCP induced more production of ROS, significantly increased DNA fragmentation, and apoptotic death than natural Cur *in vitro*. Overall, our data demonstrated that the SLCP has greater potency to kill cultured GBM cells than Cur.

The major concern regarding Cur therapy in GBM is its poor solubility, rapid degradation, and limited bioavailability as reported by several investigators [21, 27], which may limit the efficacy of natural Cur for treating GBM. In the last few years, we have been using an optimized formula of Cur (a solid lipid Cur particle, S1), to increase its bioavailability and theranostic values in different neurological diseases [24, 28, 36, 58–62]. Interestingly, we and others have found that SLCPs enter cells *in vitro* [28] and cross the blood-brain barrier readily when administered intraperitoneally in rodent [36] and in human clinical trials of Alzheimer's disease than does Cur [30]. Given this, we sought to understand the mechanisms of Cur efficacy in GBM cell lines, by comparing SLCPs to Cur as a means of developing a more effective therapy for this devastating disease. To determine the optimum dose required to attenuate GBM cell growth and proliferation, we performed MTT assays with Cur and/or SLCP, using different concentrations (1–100 *μ*M) and durations (24–72 h). We found that only the higher concentrations (>10 *μ*M) of either Cur or SLCP caused significant declines in cell viability (S2). These findings were supported by several other studies, as lower concentrations of Cur may protect cells by reducing lipid peroxidation and cytochrome-c release, whereas higher concentrations provoke GSH depletion and caspase-3 activation which induce cell death [9, 47]. We have selected 25 *μ*M of Cur or SLCP to characterize the degree of cell death, because we found that a lethal dose 50 (LD50) for Cur or SLCP was in between 25 and 50 *μ*M. When we analyzed our data, we observed that cell viability was significantly lower in SLCP-treated cells in both U-87MG and GL261 cell lines (Figures 1(a) and 1(b)).

Extrapolating these results, we have assessed whether or not Cur or SLCP selectively kills cultured GBM cells, without affecting normal cells. To this end, the neuronal cell line (SH-SY5Y) developed from human cortical tissue was treated with the same concentrations (25 *μ*M) of Cur and/or SLCP, and we observed <5% cell death in both cases, indicating that Cur had a minimal effect on neuronal cell line but induced cell death on cancer cells [47, 48]. An explanation as to why Cur or SLCP kills only tumor cells (like GBM) and not normal cells (neurons) is not yet understood, but several mechanisms have been proposed by several investigators, such as (i) cellular uptake of Cur is higher in tumor cells than in normal cells [47]; (ii) reduced glutathione (GSH) levels are lower in tumor cells than normal cells, thus enhancing the sensitivity of tumor cells to Cur [48]; and (iii) most tumor cells, but not normal cells, express constitutively active NF-*κ*B, which mediate their survival [49], whereas Cur can suppress the survival and proliferation of tumor cells by inhibiting NF-*κ*B-related signaling pathways [10].

To understand the mechanism of cell death, we have performed TUNEL staining, which identifies the DNA-fragmented cells, one of the gold standards to study cell death [50]. Many more TUNEL-positive cells were observed after treatment with SCLP than Cur at all the time points investigated (Figure 2), similar to the results of our MTT assay (Figure 1). Because TUNEL staining cannot confirm the mode of cell death, as DNA fragmentation may have occurred in the case of necrosis or apoptosis, we, therefore, performed annexin-V staining, which can differentiate the apoptotic death from necrosis [51]. Interestingly, we observed that SLCP induced more apoptosis and necrosis in comparison to Cur-treated cells, which correlated with the cell viability and TUNEL staining data (Figures 1 and 3), described above. The current study also investigated the morphology of nuclei using two different dyes, PI and Hoechst 33342, and the number of nuclear lobes caused by DNA fragmentation was counted. We found that SLCP significantly increased fragmented nuclear lobes in comparison to Cur-treated cells after 24 h and 48 h (Figure 4), indicating that SLCP induced greater DNA fragmentation than Cur in U-87MG cells. Furthermore, we also performed SCGE or comet assay, which is considered one of the gold standards for the studying the degree of DNA fragmentation *in vitro* [52]. The comet assay correlated with the TUNEL staining results described above, which confirmed the greater induction of cell death by SLCP than Cur **(**Figure 5**)**. In addition, based on the head and tail fluorescent intensity, fragmented DNA tail length, and tail moment length, we found that the % DNA in tail, extended tail length, and olive tail length were significantly higher in the case of SLCP-treated cells when compared to those cells treated with Cur (Figures 6(c), 6(d), 6(e), 6(f), and 6g), indicating SLCP has greater efficiency to damage the DNA than Cur. In addition, to confirm and support our TUNEL and comet assay results, we also performed DNA gel electrophoresis after 24 h of Cur or SLCP treatment in U-87MG cells. We observed that SLCP-treated cells produce many lower fragmented DNA bands, ranging from 180 to 100 kb or less (nucleotide oligomers) which was not seen in the case of Cur-treated cells or control groups (Figure 5(d)), confirming that SLCPs induced greater DNA fragmentation than Cur.

Several factors are involved in DNA fragmentation and cell death after treatment with Cur or SLCP, including oxidative stress [10]. We measured total ROS levels using CellROX assay after treatment of Cur or SLCP [9, 11] and observed that SLCP induced greater ROS production after 24 and 48 h of incubation than Cur (Figure 7). Excess ROS production can cause the release of apoptosis-inducing factor (AIF) from the mitochondria to the cytosol and nucleus, and activates caspase 3, thus inducing apoptosis [53]. Similarly, our Western blot results showed that activated caspase-3 (Cas-3) and Bax were significantly higher in SLCP-treated cells than Cur, whereas Bcl_2_ and c-Myc were less in SLCP-treated cells (Figures 8 and 9). Indeed, Bax and Bcl-2 play a dominant role in determining cellular fate [54], as Bcl-2 inhibits apoptosis by stabilizing the mitochondrial membrane potential [55], whereas increased expression of Bax can induce apoptosis through the release of ‘cytochrome c' from the mitochondria [56]. We found a significant upregulation of Bax and caspase-3 proteins and a downregulation of Bcl_2_ protein by SLCP treatment, relative to treatment with Cur, suggesting SLCP can induce more apoptotic death than Cur.

Furthermore, we have investigated the involvement of the p53 and c-Myc in Cur and/or SLCP-mediated apoptosis in U-87MG cells using Western blot and immunocytochemistry techniques. p53 is the main tumor suppressor protein which inhibits tumor growth; therefore, downregulation of p53 causes increase tumor formation, whereas upregulation of this protein prevents malignancy [57]. In our study, we observed a significant upregulation of p53 protein by SLCP treatment than by Cur, indicating tumorigenesis was prevented by SLCP to a greater extent than by Cur (Figures 9(a) and 9(b)). Similarly, c-Myc is another carcinogenic marker in cell [58], and activation of c-Myc leads to the unregulation of many genes, some of which are involved in cell proliferation, which can develop cancer [58]. Interestingly, we found a significant decline of c-Myc levels in SLCP-treated cells in comparison to the Cur-treated and Cur-untreated groups (Figures 9(c) and 9(d)), which again confirms that SLCP has greater antiproliferative and anticarcinogenic effects than Cur.

## 5. Conclusion

Collectively, our data suggests that Cur is a promising anticarcinogenic natural polyphenol, which has potent inhibitory properties of growth for GBM. SLCPs can induce more DNA fragmentation and can rapidly kill more GBM cells *in vitro* than Cur. SLCP-induced greater cell death is due to excess production of ROS, which increases more cell death-related proteins, reducing cell survival pathway. Taken together, our findings suggest that SLCPs can be used to treat GBM more effectively than natural Cur. Although in the present study we have shown the greater anticancer effects by SLCP than Cur in culture cell lines, we should take into consideration that the mechanistic details of GBM development, proliferation, malignancies, and metastasis in human brain are much more complex than GBM cell culture models. Therefore, a better understanding of the mechanisms of SLCP-induced GBM cell death requires further validation in animal and clinical studies, which can increase the prospects for the future treatment of this deadly malignancy.

## Supplementary Material

S1. Schematic diagram of SCLP composition and comparison of permeability in different cell lines with Cur. A: Schematic diagram showing that the Cur was coated in a solid lipid core and covered with lipid bilayer. B (Upper): solubility of Cur and SLCP in PBS. Note that most Cur particles were insoluble (crystal) in PBS, whereas SLCP became readily soluble. Middle and Lower panels: Permeability of Cur and SLCP in N2a, mouse primary hippocampal neuron (E16) and U-87MG cells after 3-, 2- and 24-h of their incubation, respectively. Note that SLCP became more permeable to those neurons and GBM cells than Cur, as indicated by more green fluorescence. Scale bar indicates 100 µm and is applicable to all the images. S2. Cell viability and morphological changes after treatment with different concentration of Cur and or SLCP. U-87MG were grown in EMEM and pen/strep (1µg/ml) for 24 h and then treated with different concentrations (1-100 µM) of either Cur or SLCP for 24 h. The images were taken by inverted phase contrast microscope (Olympus, Japan) using 10x objective. A: Cell viability was not significantly change in lower concentrations (1-5 µM) of Cur or SLCP treatment. B: Cell viability was significantly lower with 10- and 50-µM of SLCP, in comparison to Cur-treated cells. C: Morphology showed there was more cell death with SLCP-treated cells, in comparison to Cur-treated cells in all the concentration mentioned. Scale bar indicates 100 µm. ∗p<0.05 and ∗∗p<0.01 compared to Cur-treated cells.

## Figures and Tables

**Table 1:** Sources of different antibodies used in this study.

Antibodies	Source	Type	Company	Catalog number	Address
Caspase-3	Rabbit	Polyclonal	Cell Signaling Technology	9661	Danvers, MA
Bax	Rabbit	Polyclonal	Cell Signaling Technology	2772	Danvers, MA
Bcl_2_	Mouse	Monoclonal	Santa Cruz Biotech	Sc-7382	Santa Cruz, CA
Akt	Rabbit	Monoclonal	Cell Signaling Technology	9272S	Danvers, MA
pAkt (Ser473)	Rabbit	Monoclonal	Cell Signaling Technology	9271	Danvers, MA
p53	Rabbit	Polyclonal	Cell Signaling Technology	9282	Danvers, MA
c-Myc	Rabbit	Polyclonal	Cell Signaling Technology	9402	Danvers, MA
*β*-Tubulin	Rabbit	Monoclonal	Cell Signaling Technology	2128	Danvers, MA

## Abbreviations

Cur: Curcumin
SLCP: Solid lipid curcumin particles
GBM: Glioblastoma
MTT: 3-(4,5-Dimethylthiazol-2-yl)-2,5-diphenyltetrazolium bromide
TUNEL: Terminal deoxyribonucleic acid nick end labeling
ROS: Reactive oxygen species
Bax: Bcl_2_-associated X protein
Bcl_2_: B-cell lymphoma 2
TMZ: Temozolomide
WHO: World Health Organization
SCGE: Single-cell gel electrophoresis
PI: Propidium iodide
AD: Alzheimer's disease
mTOR: Mechanistic target of rapamycin
NF-*κ*B: Nuclear factor kappa beta
Jak1: Janus kinase-1
STAT3: Signal transducer and activator of transcription 3
SLP: Solid lipid particle
ATCC: American type cell culture
EMEM: Eagle's Minimum Essential Medium
FBS: Fetal bovine serum
E16: 15 embryonic-16
HBSS: Hank's balanced salt solution
SEM: Standard error of mean
BrdU: Bromo uridin
ex/em: Excitation/emission
PBS: Phosphate buffer saline
DPBS: Dulbecco's phosphate buffer saline
EDTA: Ethylene-di-amino-tetra-acetic-acid
DCFH: DA-dichloro-dihydro fluorescein diacetate
rpm: Revolution per minute
mM: Millmolar
RIPA: Radio immunoprecipitation assay
EGTA: Ethylene glycol tetra acetic acid
SDS: Sodium dodecyl sulfate
BCA: Bicinchoninic acid assay
PVDF: Polyvinylidene fluoride
HRP: Horseradish peroxidase
ANOVA: One-way analysis of variance
HSD: Honestly significant difference
*μ*M: Micromolar
GSH: Reduced glutathione
AIF: Apoptotic inducing factor
AU: Arbitrary unit
OD: Optical density
SDS-PAGE: Sodium dodecyl sulfate polyacrylamide gel electrophoresis
TBS: Tris buffer saline.
