# Supplementary material for: Solid Lipid Curcumin Particles Induce More DNA Fragmentation and Cell Death in Cultured Human Glioblastoma Cells than Does Natural Curcumin
Source: Oxid Med Cell Longev. 2017 Nov 19;2017:9656719. doi: 10.1155/2017/9656719 (PMC5735327; doi:10.1155/2017/9656719)
Supplement: Supplementary file 1 — S1. Schematic diagram of SCLP composition and comparison of permeability in different cell lines with Cur. A: Schematic diagram showing that the Cur was coated in a solid lipid core and covered with lipid bilayer. B (Upper): solubility of Cur and SLCP in PBS. Note that most Cur particles were insoluble (crystal) in PBS, whereas SLCP became readily soluble. Middle and Lower panels: Permeability of Cur and SLCP in N2a, mouse primary hippocampal neuron (E16) and U-87MG cells after 3-, 2- and 24-h of their incubation, respectively. Note that SLCP became more permeable to those neurons and GBM cells than Cur, as indicated by more green fluorescence. Scale bar indicates 100 µm and is applicable to all the images. S2. Cell viability and morphological changes after treatment with different concentration of Cur and or SLCP. U-87MG were grown in EMEM and pen/strep (1µg/ml) for 24 h and then treated with different concentrations (1-100 µM) of either Cur or SLCP for 24 h. The images were taken by inverted phase contrast microscope (Olympus, Japan) using 10x objective. A: Cell viability was not significantly change in lower concentrations (1-5 µM) of Cur or SLCP treatment. B: Cell viability was significantly lower with 10- and 50-µM of SLCP, in comparison to Cur-treated cells. C: Morphology showed there was more cell death with SLCP-treated cells, in comparison to Cur-treated cells in all the concentration mentioned. Scale bar indicates 100 µm. ∗p<0.05 and ∗∗p<0.01 compared to Cur-treated cells. [file 9656719.f1.docx]

**Supplemental figures**

**
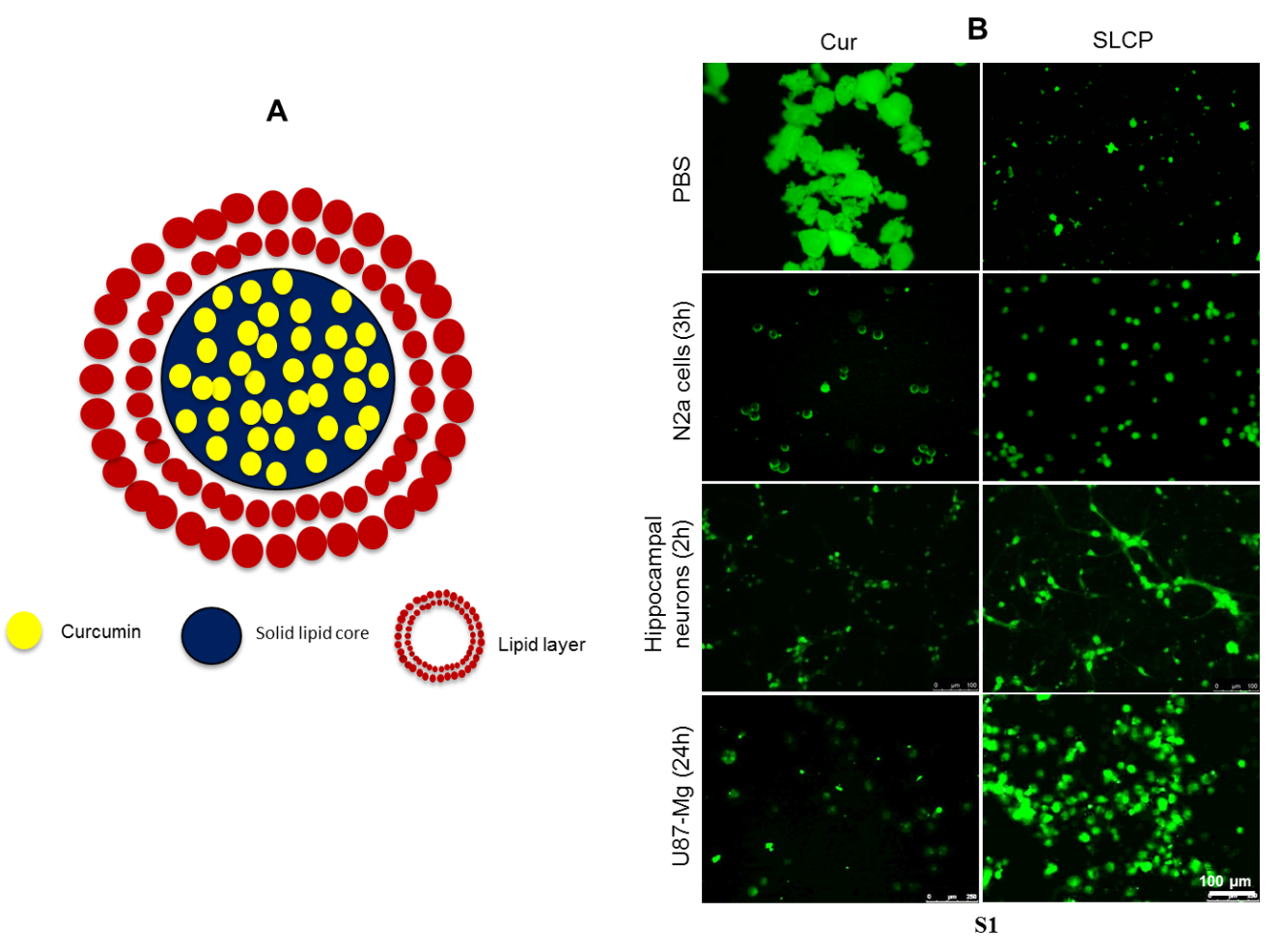
**

**S1. Schematic diagram of SCLP composition and comparison of permeability in different cell lines with Cur. A**: Schematic diagram showing that the Cur was coated in a solid lipid core and covered with lipid bilayer. **B** (**Upper):** solubility of Cur and SLCP in PBS. Note that most Cur particles were insoluble (crystal) in PBS, whereas SLCP became readily soluble. **Middle and Lower panels:** Permeability of Cur and SLCP in N2a, mouse primary hippocampal neuron (E16) and U-87MG cells after 3-, 2- and 24-h of their incubation, respectively. Note that SLCP became more permeable to those neurons and GBM cells than Cur, as indicated by more green fluorescence. Scale bar indicates 100 µm and is applicable to all the images.

**
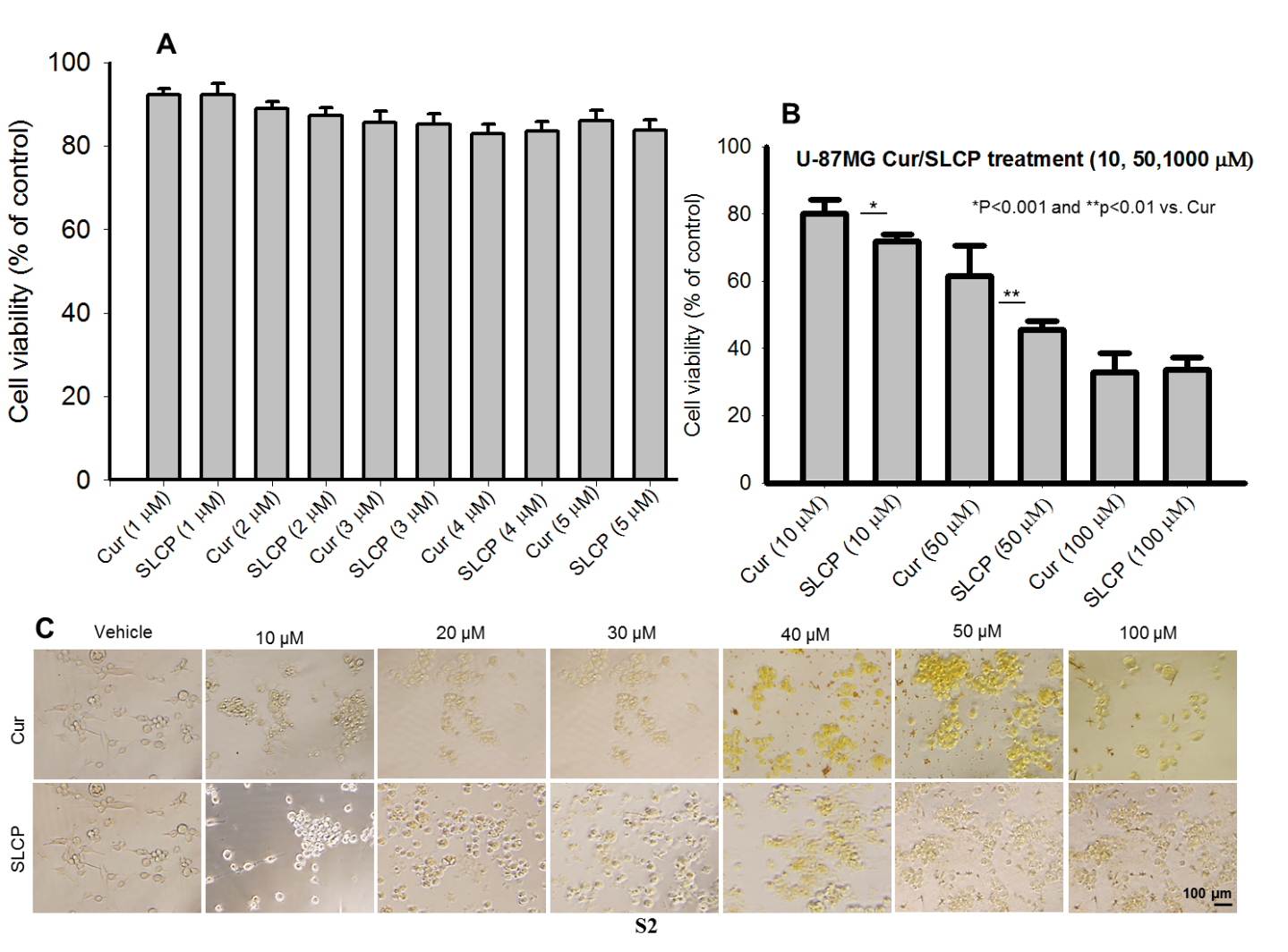
**

**S2. Cell viability and morphological changes after treatment with different concentration of Cur and or SLCP.** U-87MG were grown in EMEM and pen/strep (1µg/ml) for 24 h and then treated with different concentrations (1-100 µM) of either Cur or SLCP for 24 h. The images were taken by inverted phase contrast microscope (Olympus, Japan) using 10x objective. **A**: Cell viability was not significantly change in lower concentrations (1-5 µM) of Cur or SLCP treatment. **B**: Cell viability was significantly lower with 10- and 50-µM of SLCP, in comparison to Cur-treated cells. **C**: Morphology showed there was more cell death with SLCP-treated cells, in comparison to Cur-treated cells in all the concentration mentioned. Scale bar indicates 100 µm. *p<0.05 and **p<0.01 compared to Cur-treated cells.
